# The MRZ reaction helps to distinguish rheumatologic disorders with central nervous involvement from multiple sclerosis

**DOI:** 10.1186/s12883-018-1018-3

**Published:** 2018-01-31

**Authors:** Tilman Hottenrott, Rick Dersch, Benjamin Berger, Dominique Endres, Daniela Huzly, Jens Thiel, Sebastian Rauer, Oliver Stich, Ulrich Salzer, Nils Venhoff

**Affiliations:** 1 Department of Neurology and Neurophysiology, Faculty of Medicine, Medical Center – University of Freiburg, Breisacher Strasse 64, D-79106 Freiburg, Germany; 2 Department of Psychiatry and Psychotherapy, Faculty of Medicine, Medical Center - University of Freiburg, Hauptstraße 5, D-79104 Freiburg, Germany; 3 Institute of Virology, University Medical Center Freiburg, Hermann-Herder-Strasse 11, D-79104 Freiburg, Germany; 4 Department of Rheumatology and Clinical Immunology, Faculty of Medicine, Medical Center – University of Freiburg, Hugstetter Strasse 55, D-79106 Freiburg, Germany

**Keywords:** Neuropsychiatric systemic lupus erythematosus (NPSLE), ANCA-associated vasculitides, Behçet’s disease, Multiple sclerosis (MS), Intrathecal polyspecific antiviral immune response, MRZ reaction (MRZR)

## Abstract

**Background:**

Some rheumatologic disorders may initially manifest with central nervous system (CNS) affection, mimicking the clinical, magnetic resonance imaging, and cerebrospinal fluid findings of multiple sclerosis (MS). The MRZ reaction (MRZR), composed of the three respective antibody indices (AIs) against measles, rubella, and varicella zoster virus, has been found positive frequently in MS patients. However, it is unclear whether the MRZR is helpful to distinguish rheumatologic disorders with CNS involvement (RDwCNS) from MS.

**Methods:**

The MRZR was evaluated in patients with RDwCNS (*n* = 23), MS (*n* = 46; age and sex matched to patients with RDwCNS), and other inflammatory autoimmune neurological diseases affecting the CNS (OIND; *n* = 48). Both the stringency levels that have been used in previous MRZR studies, MRZR-1 (≥ 1 of 3 AIs positive) and MRZR-2 (≥ 2 of 3 AIs positive), were applied.

**Results:**

There was no statistically significant difference in the prevalence of positive MRZR between patients with RDwCNS (MRZR-1: 13.0% and MRZR-2: 8.7%, respectively) and OIND (MRZR-1: 22.9% and MRZR-2: 8.3%, respectively). Compared to these two study cohorts, the MS group exhibited significantly higher prevalences of positive MRZR (MRZR-1: 82.6%, MRZR-2: 63.0%; *p* < 0.005 each).

**Conclusions:**

Considering the high specificity of MRZR-2 for MS found in this study, MRZR-2 can be a useful diagnostic tool for distinguishing MS from RDwCNS or OIND.

## Background

Some rheumatologic disorders may initially present with central nervous system (CNS) involvement, mimicking the clinical, magnetic resonance imaging (MRI), and cerebrospinal fluid (CSF) findings of multiple sclerosis (MS).

In connective tissue diseases such as systemic lupus erythematosus (SLE), neuropsychiatric manifestations (NPSLE) are found in up to 66% patients and mostly affect the CNS [1]. Other central NPSLE, including demyelinating syndromes (e.g. optic neuritis) or transverse myelitis may also occur [2]. In addition, NPSLE develops in 28%–40% of patients before or around the time of the initial diagnosis of SLE [2]. Although several studies have reported different pathways for explaining the pathophysiology of NPSLE, the exact underlying mechanisms are still unclear [2]. The intrathecal synthesis of antibodies and the migration of T cells, B cells, macrophages, and monocytes across the blood-brain barrier into the CNS were observed [1]. These mechanisms potentially result in CSF alterations (mild pleocytosis in 30%–40% [2] or elevated immunoglobulin G (IgG) indices in up to 75% [2], and MRI findings (inflammatory T2 lesions in 40% [3]) similar to those in MS patients. Thus, NPSLE can be difficult to distinguish from MS; this is also true for other rheumatologic disorders with CNS involvement (RDwCNS) such as primary vasculitides, including granulomatosis with polyangiitis (GPA, Wegener’s granulomatosis) [4, 5], microscopic polyangiitis (MPA) [6], and Behçet’s disease [7]. For several reasons the reliable differentiation between SLE and MS is important, at least because of significant differences in treatment regimens [2, 8].

The MRZ reaction (MRZR) is a polyspecific, intrathecal humoral immune response directed against the three neurotropic viruses: measles (M), rubella (R), and varicella zoster (Z), assessed using the three respective antibody indices (AIs) [9]. Several studies have shown a high prevalence of positive MRZR in the majority of relapsing-remitting MS (RRMS) [10] and primary progressive MS (PPMS) patients [11]. The pathophysiological role of MRZR currently remains unclear; the most important role of MRZR in clinical practice is its potential to establish a differential diagnosis of MS because a positive MRZR appears to be highly specific for MS [10]. MRZR was negative in healthy subjects [12]; in most patients with infectious neurological disorders [13, 14]; and in the majority of patients with other inflammatory autoimmune neurological diseases (OIND), including neuromyelitis optica [15], paraneoplastic neurological syndromes [16], neurosarcoidosis, and acute disseminated encephalomyelitis [17].

However, to our knowledge, the prevalence of positive MRZR in RDwCNS patients has not been systematically studied; only few case series with a small sample size have been reported on this subject [10]. Consequently, whether MRZR can be helpful in distinguishing MS from RDwCNS is unclear. Therefore, this study was planned with the objective of systematically assessing and comparing the prevalence of positive MRZR in a well-defined cohort of RDwCNS patients with that in MS and OIND patients.

## Methods

### Patients

This was a retrospective study that enrolled patients treated at the University Medical Centre Freiburg in Germany between 2005 and 2016, using an electronic database search. Lumbar puncture (LP) had already been performed in all patients for clinical purposes after obtaining written consent. Paired CSF and serum samples were collected on the same day and stored according to the consensus protocol for the standardization of CSF collection and biobanking [18]. Haemolytic CSF samples were excluded.

Patients with RDwCNS had been diagnosed by board certified rheumatologists of the University Medical - Centre Freiburg. Systemic lupus erythematosus (SLE) was identified according to the 2007 revised criteria for the classification of SLE [19]; ANCA associated vasculitides, both, MPO-associated MPA and PR3-associated GPA (Wegener’s granulomatosis) according to the revised international Chapel Hill consensus conference nomenclature of vasculitides [20, 21], and Behçet’s disease according to the International Study Group for Behçet’s disease [22]. CNS involvement of RDwCNS was defined on the basis of clinical signs such as headache, neuropsychological disturbances or focal neurological disturbances (all without better explanation), and the presence of either of the following two paraclinical findings: (1) inflammatory changes in the CSF, such as elevated cell count, intrathecal immunoglobulin synthesis, positive oligoclonal bands (OCB), or significant disturbance in the blood-CSF barrier indicated by an age-related elevation in the albumin quotient or (2) inflammatory signs in brain or spinal MRI compatible with RDwCNS as assessed by neuroradiologists of the University Medical - Centre Freiburg. Patients with suspected RDwCNS were excluded if they fulfilled the 2010 revised McDonald criteria for MS [23]. All patients with RDwCNS who fulfilled these criteria were enrolled in this study.

Diagnosis of MS was established according to the 2010 revised McDonald criteria with particularly careful exclusion of the relevant differential diagnoses [23]. MS patients were drawn from a cohort of MS patients (comprising 103 patients with PPMS and 100 with RRMS) from an earlier study [11]. A previously established in-house matching software was used to select the MS patients for this study who best matched for age and sex with the RDwCNS patients in a 2:1 (MS:RDwCNS) ratio [24].

As the third study group, forty-eight patients with OIND, for whom MRZR test results were available from previous research [17], were enrolled. Data concerning the ethnicity and immunization status of study patients were not available. This study was approved by the ethics committee of the University Medical Centre Freiburg (EK-Fr 489/14).

### Materials and methods

MRZR was analysed at the Department of Virology of the University of Freiburg. All routine CSF measurements were carried out in the CSF laboratory of the University Medical Centre Freiburg. Total immunoglobulin concentrations in the serum and the CSF were detected nephelometrically (ProSpect System, Siemens, Germany), while measles-, rubella- and varicella-IgG (IgGspec) levels in the CSF and the serum were measured using enzyme-linked immunosorbent assay (Serion *classic* ELISA, Germany). MRZR was determined from the three respective virus-specific AIs that were calculated as follows: AI (antibody index) = QIgG[spec]/QIgG[total], if QIgG[total] < Qlim, and AI = QIgG[spec]/Qlim, if QIgG[total] > Qlim [25]. The upper reference range of QIgG, Qlim, was calculated according to Reiber’s formula [25]. For a positive AI result indicative of intrathecal IgG production against the respective pathogen, a threshold of ≥1.5 was applied. Most previous studies have varied as to how many positive AIs are required for a positive MRZR [11]. In this study, MRZR-2 was defined as that with two or three positive AIs, and MRZR-1 as that requiring only one or more positive AIs. In cases where an AI could not be calculated because no antibodies were detected in the CSF, the AI was considered as 1.0 (negative).

As the focus in this study was on RDwCNS, additional data of these patients regarding the following were obtained: (1) results of brain/spinal MRI performed and routinely assessed at the Department of Neuroradiology of the University Medical - Centre Freiburg from medical records, (2) results of the CSF routine test (including the parameters of total cell count, age-related albumin quotient, quantitative intrathecal antibody synthesis, and OCB) from medical records, and (3) the AIs of four characteristic autoantibodies (dsDNA, Cardiolipin, PR3, and MPO). The serum samples of RDwCNS patients were therefore initially screened for the presence of autoantibodies associated with rheumatologic disorders if sufficient serum sample was available after the routine examinations and the MRZR measurements. Anti-nuclear antibody (ANA) staining pattern was assessed using indirect immunofluorescence (IIF) on 2100-Ro HEp-2000® cells (Fluorescent ANA-RoTest System, Immuno Concepts N.A. Ltd., Sacramento, CA, USA). RDwCNS Patients with positive IIF were screened for antibodies using enzyme-linked immunosorbent assay (ELISA) directed against extractable nuclear antigens (ENA) using ANA Profil 3 (DL1590–3 G, EUROIMMUN AG, Luebeck, Germany). Antibodies against double stranded deoxyribonucleic acid (DNA) were detected using dsDNA IgG ELISA (212,196, Euro Diagnostica AB, Malmö, Sweden). Phospholipid antibodies were measured using Cardiolipin IgG ELISA (212,796, Euro Diagnostica AB, Malmö, Sweden). ANCA specificity for PR3 (Orgentec Diagnostika GmbH, Mainz, Germany) or myeloperoxidase (MPO) (Euroimmun, Medizinische Labordiagnostika AG, Luebeck, Germany) was measured using ELISA as well. Assessment was done according to the manufacturers’ reference ranges with the upper normal limit of 40 U/mL for dsDNA, 14 U/mL for Cardiolipin, 10 U/mL for PR3, and 20 U/mL for MPO. In case of an elevated concentration of antibodies against dsDNA, Cardiolipin, PR3, or MPO in serum, the CSF titre was additionally measured for calculating the respective AI, as described for MRZR above.

The detection of OCB was performed using an isoelectric focusing technique on agarose gel followed by immunofixation (Hydragel Isofocusing, Sebia, France). A positive OCB result was defined as two or more OCB [26].

### Statistical analyses

Statistical testing of the differences between the three study groups with respect to sex, the prevalence of positive single MRZ-AIs, the prevalence of positive MRZR and OCB was performed using Fisher’s exact test (two-tailed). Differences of the mean age between the groups were tested using Student’s t-test (two-tailed) as this data was consistent with a normal distribution according to the Kolmogorov-Smirnov test. The other metric items (mean AI and mean disease duration) were compared with the Mann-Whitney U test (two-tailed) as their data was not normally distributed according to the Kolmogorov-Smirnov test. A possible correlation between the disease duration (defined as the time interval between clinical onset and the time of LP) and the MRZR status was investigated using the point biserial correlation analysis (two-tailed). A *p*-value < 0.05 was regarded as statistically significant.

## Results

### Study population

Fifty patients diagnosed with RDwCNS were retrospectively screened to confirm the diagnosis and for the availability of sufficient clinical, CSF, and MRI data as well as adequate CSF/serum samples for the determination of MRZR. Twenty-seven patients had to be excluded because of missing CSF/serum samples. Consequently, the RDwCNS group comprised 23 well-characterized patients, including 20 patients with connective tissue disease (18 with SLE and two with an undifferentiated connective tissue disease (UCTD)) and three patients with primary vasculitides (one MPO-associated MPA, one PR3-associated GPA, and one Behçet’s disease). For comparison, a sample of 46 MS patients (31 with RRMS and 15 with PPMS) matched for age and sex to RDwCNS patients was drawn. The OIND group comprised 22 patients with neurosarcoidosis, 19 with autoimmune encephalitis, and 7 with acute disseminated encephalomyelitis. Table 1 shows the key demographic features of the three study cohorts.

**Table 1:** Demographic data

Study group	RDwCNS (n = 23)	MS (*n* = 46)	OIND (*n* = 48)	Comparison statistics
Sex, Females in %	78.3	78.3	41.7	RDwCNS vs. MS: n.s. Both groups vs. OIND: *p* < 0.01.
Mean age in years at the time of LP (range; SD)	44.8 (21–75; 16.8)	43.9 (20–74; 15.6)	51.8 (4–84; 18.4)	All comparisons: n.s.
Disease duration at the time of LP in months (range; SD)	77.0 (0–516; 112.6)	60.5 (0–468; 91.1)	11.6 (0–120; 24.7)	RDwCNS vs. MS: n.s. Both groups vs. OIND: p < 0.005

*RDwCNS* Rheumatologic disorders with CNS involvement, *MS* Multiple sclerosis, *OIND* Other inflammatory autoimmune neurological diseases [17], *LP* Lumbar puncture, *SD* Standard deviation, *n.s* Not significant

### Virus-specific antibody indices

Detailed results of the MRZ-AIs in the three study groups are shown in Table 2. No statistically significant differences were found between the RDwCNS and OIND patients concerning the frequency of positivity and the mean values of any of the three AIs. Compared to both of these study cohorts, the MS group showed a significantly higher frequency of positive AIs for each virus and higher mean AI values for all three viruses.

**Table 2:** Frequencies of positive antibody indexes (AIs) for MRZ

Study group	RDwCNS(n = 23)	MS(n = 46)	OIND (n = 48)	Comparison statistics
Patients with 0 positive AI	87.0%	17.4%	77.1%	RDwCNS vs. OIND: n.s. Both groups vs. MS: *p* < 0.0001.
Patients with 1 positive AI	4.3%	19.6%	14.6%	All comparisons: n.s.
Patients with 2 positive AIs	8.7%	28.3%	6.3%	Both groups vs. RDwCNS: n.s. OIND vs. MS: p < 0.01
Patients with 3 positive AIs	0%	34.8%	2.1%	RDwCNS vs. OIND: n.s. Both groups vs. MS: *p* < 0.001.
Positive AIs for M	8.7%	63.0%	6.3%	RDwCNS vs. OIND: n.s. Both groups vs. MS: p < 0.0001.
Positive AIs for R	4.3%	63.0%	12.5%	RDwCNS vs. OIND: n.s. Both groups vs. MS: p < 0.0001.
Positive AIs for Z	8.7%	54.3%	14.6%	RDwCNS vs. OIND: n.s. Both groups vs. MS: p < 0.0001.
Mean AI values for M (range; SD)	1.1 (0.6–2.0; 0.3)	4.0 (0.6–52.2; 7.6)	1.0 (0.6–2.6; 0.3)	RDwCNS vs. OIND: n.s. Both groups vs. MS: p < 0.0001.
Mean AI values for R (range; SD)	1.1 (0.6–3.6; 0.6)	3.4 (0.7–19.8; 4.2)	1.2 (0.6–8.3; 1.2)	RDwCNS vs. OIND: n.s. Both groups vs. MS: p < 0.0001.
Mean AI values for Z (range; SD)	1.2 (0.6–4.2; 0.7)	3.9 (0.6–25.4; 5.2)	1.2 (0.4–3.8; 0.6)	RDwCNS vs. OIND: n.s. Both groups vs. MS: p < 0.01.

*RDwCNS* Rheumatologic disorders with CNS involvement, *MS* Multiple sclerosis, *OIND* Other inflammatory autoimmune neurological diseases [17], *SD* Standard deviation; *positive AI* Antibody index for measles (M), rubella (R) or varicella zoster (Z) ≥ 1.5; *n.s* Not significant

### MRZR

A positive MRZR was found in a minority of the patients with RDwCNS (MRZR-2: 8.7% and MRZR-1: 13.0%) and OIND (MRZR-2: 8.3% and MRZR-1: 22.9%); the differences in both the MRZR definitions between these two cohorts were statistically not significant. The only two MRZR-2 positive RDwCNS patients were both female. The first patient was a 45-year-old woman diagnosed with SLE with CNS involvement. She had developed neuropsychological disturbances (impaired concentration and memory as well as mild aphasia), hemihypesthesia, and headache. Abnormal diagnostic parameters obtained at the time of the first diagnosis included positive ANA (1:400) with specificity against dsDNA (154 U/mL) in serum; lowered C3 and C4 along with increased C3d in the serum; inflammatory CSF changes (elevated cell count of 7/μL, intrathecal IgG synthesis, and positive OCB exclusively in the CSF). Her brain MRI displayed a noticeable amount of multiple, predominantly peripherally located subcortical T2 lesions without contrast enhancement that were found to be compatible with CNS affection of SLE. The second MRZR-2 positive RDwCNS patient was 75 years old at the time of the first diagnosis with an UCTD. The first neurological symptom had occurred thirteen years prior with unilateral facial palsy accompanied by intrathecal IgG synthesis and positive OCB in the CSF. Ten years after this, gradually, progressive paraparesis with gait disturbance, myalgia, and headache occurred. Comprehensive diagnostic work-up revealed highly positive ANA (1:3200) with anti-centromere specificity in ENA differentiation, persisting intrathecal IgG synthesis, and positive OCB in the CSF with multiple infra- and supratentorial subcortical T2 lesions without contrast agent enhancement in the MRI of the brain and the spinal cord that were assessed as chronic inflammatory features. No clinical signs of the limited cutaneous form of systemic sclerosis were found.

**Fig. 1:**
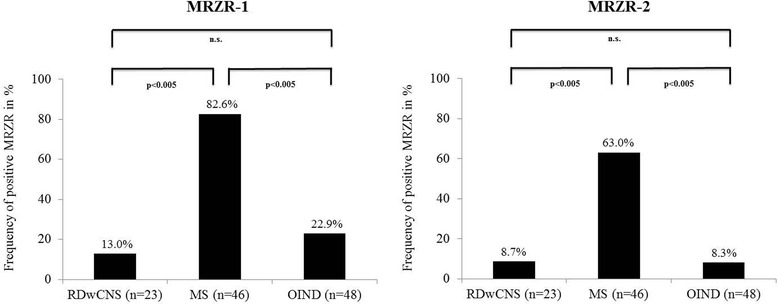
Frequency of positive MRZR-1 (≥ 1 positive AI) and MRZR-2 (≥ 2 positive AIs) in patients with rheumatologic disorders with involvement of the central nervous system (RDwCNS), multiple sclerosis (MS), and other inflammatory autoimmune neurological diseases (OIND; [17])

In contrast to the RDwCNS and OIND patients, the MS patients showed a significantly higher prevalence of positive MRZR regardless of the definition used (MRZR-2: 63.0% and MRZR-1: 82.6%; *p* < 0.005 for the comparisons of MRZR-1 and MRZR-2 with those of the RDwCNS and OIND groups; Fig. 1). By using a higher threshold of ≥2.0 for a positive AI, the prevalence of positive MRZR-2 dropped to 0% in the RDwCNS group, to 4.1% in the OIND group, and to 47.8% in the MS group (p < 0.005 for each comparison with the MS group).

There was no significant correlation between the disease duration and the MRZR result of patients in all three study groups (RDwCNS: r_pb_ − 0.19; MS: r_pb_ + 0.18; OIND: r_pb_ + 0.19; *p* > 0.05 for all groups).

### Routine CSF findings of patients with RDwCNS

The majority of patients with RDwCNS (78.3%) showed inflammatory CSF signs, while the others (21.7%) had completely normal routine CSF results. The most frequent pathological findings were an elevated total CSF cell count (43.5%) (median cell count of those patients with pleocytosis: 9.5/μL; range: 6–433/μL; SD: 126.5), an elevated albumin quotient (43.5%), OCB (39.1%), and the intrathecal synthesis of IgG (21.7%), immunoglobulin (Ig) M (17.4%), or IgA (13.0%). Positive OCB were found in 93.5% of the MS patients and in 31.3% of OIND patients.

### Autoantibodies

Twenty-one patients with RDwCNS, including 19 patients with connective tissue disease (CTD) (17 with SLE and two with UCTD) and two patients with primary vasculitides (one with MPA and one with GPA) were screened for autoantibodies. All CTD patients had at least once a positive detection of ANA in their medical history. At the time of the CSF analyses, 15 of the 19 CTD patients (78.9%) still had an increased ANA serum concentration. Thirty-six MS patients were screened for ANA and 50.0% showed a positive result. With regards to ENA differentiation, CTD patients revealed antibodies against dsDNA or anti-snRNP/Sm-antibodies in 6/19 patients (31.6%), anti-nucleosome antibodies in 6/19 (31.6%), anti-Ro-52 and anti-SS-A/Ro in 4/19 (21.1%), and anti-SS-B/La in 3/19 (15.8%) patients. All other ENA subspecificities were present in less than two subjects; 4/19 CTD patients (21.1%) did not show any positive ENA autoantibody. Cardiolipin antibodies were positive in 11/19 patients (57.9% of CTD patients). Both patients with ANCA-associated vasculitis had a positive ANCA in IIF with specificity for MPO in one (with MPA) and for PR3 in the other case (with GPA).

In patients with positive autoantibody detection in serum, we found a positive AI for anti-cardiolipin-IgG in 2/11 patients (18.2%; exact AI values: 1.6 and 2.9), a positive AI for anti-PR3-IgG in 0/1 patient (0%), a positive AI for anti-MPO-IgG in 0/1 patient (0%), and a positive AI for anti-dsDNA-IgG in 0/4 patients (0%).

### MRI findings of patients with RDwCNS

The brain and/or spine MRI results were abnormal in 82.6% of all patients with RDwCNS. Most frequently, supratentorial, subcortical hyperintense T2 lesions without contrast enhancement compatible with vasculitis (60.9%) were found. Fewer RDwCNS patients displayed cerebral ischemia compatible with vasculitis (13.0%), T1 lesions with contrast enhancement (13.0%), brain stem T2 lesions (8.7%), cerebral atrophy (8.7%), cortical T2 lesions without contrast enhancement compatible with vasculitis (4.3%), and hydrocephalus (4.3%).

## Discussion

To the best of our knowledge, this is the first systematic investigation of MRZR in a well-characterized larger cohort of patients with RDwCNS. The main findings of our study are that patients with RDwCNS, who may exhibit clinical symptoms as well as CSF and MRI findings similar to those of MS patients, have a positive MRZR much less frequently than MS patients.

### Study population

The mean age of the subjects in all three study groups was similar; sex distribution of the OIND (that had fewer female subjects) differed from those of the other two study groups, which were matched for age and sex. Age and sex distribution of this RDwCNS cohort corresponded to the epidemiological data of German SLE patients [27]. The mean age of the MS group was unusually high for patients with RRMS [28]; this was most likely due to the fact that these MS patients were selected on the basis of their matching characteristics with RDwCNS patients. The similar age and disease duration of MS and RDwCNS patients are advantages for interpretation of their MRZR results since these items may have an effect on the prevalence of positive MRZR outcomes. Indications for this are the lower prevalence in paediatric compared to adult MS patients and increasing prevalence within the individual disease course [29, 30]. However, in the present study there was no correlation between the disease duration and the MRZR result of all patients irrespective to their diagnosis group.

### MRZ reaction

To date, only few very small case series regarding MRZR prevalence in RDwCNS patients have been conducted [10]. In the study with the most RDwCNS patients, Greaf et al. found a positive MRZR-2 in three of the nine SLE patients, in the only one with Sjögren’s syndrome, and in the only one with GPA, and concluded that MRZR is not MS-specific [31]. More recently, six patients with NPSLE and two with CNS vasculitis (Horton’s disease) were studied for MRZR-2 without a single positive result [10]. In agreement with these findings, our study also found positive MRZR-2 in only few patients with RDwCNS; however, the proportions were noticeably smaller compared to that in the study by Greaf et al. [31]. This may have been due to the relevant difference in the sample sizes. As there is no existing standard “rule-out test” for MS, it cannot be completely excluded that the two MRZR-2 positive patients diagnosed with RDwCNS might have a MS form simultaneously. Routine CSF tests and brain MRI could not adequately differentiate RDwCNS from MS in these two patients. However, in both the patients, the extra-cerebral symptoms, as well as paraclinical findings like ANA (with specificity for dsDNA and anti-centromere, respectively) and activation of the complement cascade are indicators for a CTD.

Frequencies of positive MRZR and positive AIs for measles, rubella, and varicella zoster as well as the mean AI of all three viruses did not differ between the RDwCNS and OIND patients; however, all these values were much lower in both the groups compared to that in the MS group. Consequently, these study results are another indication that a positive MRZR does not represent an unspecific sign of general CNS autoimmunity in addition to those found by Jarius et al. [15, 16]. A similar MRZR-2 positivity exhibited by the present MS group has also been found in several previous studies [9, 32–34], in a recent comprehensive MRZR review [10], and in an earlier study by our group [17]. The comparatively rare positivity of MRZR-2 in the present RDwCNS and OIND groups indicates that especially MRZR-2 might be helpful in distinguishing MS from other possible diagnoses. This corresponds to the recent MRZR review that reported an overall MRZR-2 specificity of 97% for MS [10]. The specificity of MRZR-2 in the context of the present study could be increased by using an AI threshold of > 2.0, leading to the complete absence of MRZR-2 positive RDwCNS patients in this cohort and around 50% MRZR-2 positive MS patients. MRZR-1 is clearly less specific, considering the higher number of “false positive” results in the RDwCNS and OIND patients. Therefore, we conclude that the MRZR-2 definition should be preferred.

### Additional paraclinical findings of RDwCNS patients

Results of the routine CSF analyses and MRI assessments illustrate the degree of difficulty faced in the paraclinical distinction between MS and RDwCNS. The majority of RDwCNS patients showed unspecific inflammatory changes such as a slight to moderate CSF pleocytosis, OCB in the CSF, or intrathecal Ig synthesis; all characteristics that are also compatible with MS [25]. OCB have a higher sensitivity in MS patients (93.5% in this cohort in accordance with [35]) compared to MRZR. However, they were found to be less specific, as illustrated by their prevalence of 30–40% in the present RDwCNS and OIND cohorts. This finding is similar to the results of another study on NPSLE patients who had OCB in 50% [36]. Accordingly, the more specific MRZR and the more sensitive OCB are both important and complementary components of the diagnostic work-up for MS [37]. A dysfunction of the blood-CSF barrier detected in around 40% of RDwCNS patients is not typical for MS patients, but a mild blood-CSF barrier dysfunction can be observed in about 20% in MS patients [38]. These findings prove that routine CSF parameters alone (without MRZR) are not sufficient to adequately distinguish between MS and RDwCNS [39]. This also stands true for brain MRI, supported by the results in our RDwCNS cohort, where more than 60% patients displayed supratentorial, subcortical hyperintense T2 lesions. This is in consensus with earlier findings showing periventricular or subcortical white matter T2 hyperintense lesions in 50%–75% of NPSLE patients [2]. The challenge in this context is that similar T2 lesions are also very common among MS patients [40]. MRI findings not suspicious for MS, such as cerebral ischemia or hydrocephalus, were found in only few RDwCNS subjects (< 20%). Apart from the analysis of the CSF and MRI, the detection of ANAs is usually indicative of a connective tissue disease. However, non-specific ANA can be observed in some MS patients (in around 30% according to one study and in even 50% of the present MS cohort), sometimes leading to diagnostic uncertainty [41]. In the present RDwCNS cohort, most patients had at least one positive ENA autoantibody, a more reliable indication of a rheumatologic disorder compared to ANA. Furthermore, a minority of the RDwCNS patients revealed a positive AI for the respective autoantibody detected in the serum. To our knowledge, this has never been reported before.

It needs to be acknowledged that in many RDwCNS patients, extra-cerebral clinical signs such as arthralgia, skin manifestations, or affection of internal organs (lungs, heart, or kidneys) are indicative of a systemic rheumatic disorder, not of MS. However, because neurological symptoms may signal the clinical onset of RDwCNS, differentiating MS and RDwCNS can also clinically be very challenging [39]. In every patient with a suspected inflammatory CNS affection the comprehensive evaluation of all clinical, blood and CSF biomarkers, as well as neurophysiological and MRI data is indispensable. If diagnostic uncertainty then still persists, a biopsy should be considered in case of a significantly affected patient and if the exact diagnosis is relevant to the choice of the therapeutic drug.

### Limitations

The most important limitations of this MRZR study are the monocentric, retrospective design and the small sample size of patients with RDwCNS. Further study limitations include the lack of data concerning ethnicity and vaccination status. Local infection rates and vaccination status with respect to the three MRZ viruses may have influenced the MRZR results, as has been shown for the rubella virus in Cuba [42]. Furthermore, ethnicity plays a role in the prevalence of certain disorders, e.g., Asian and Afro-Caribbean SLE patients have a higher rate of neuropsychiatric involvement than Caucasians [43–45]. Therefore, verification of the present MRZR results in a larger, prospective, multiethnic RDwCNS cohort with known vaccination status is warranted. Regarding the AIs for autoantibodies, it has to be considered that the measurement of autoantibodies in the CSF has not yet been established.

## Conclusions

MRZR-2 may be helpful in distinguishing MS from other inflammatory autoimmune disorders with primary (such as OIND) or secondary CNS affection (such as RDwCNS). Furthermore, results of the present study strengthen the significance of CSF analysis as an indispensable tool for ensuring prompt detection of MS and its differential diagnosis, enabling appropriate treatment [37].

## Abbreviations

AI: antibody index
AIs: antibody indices
ANA: anti-nuclear antibody
C3: complement component 3
C3d: complement component 3d
C4: complement component 4
CNS: central nervous system
CSF: cerebrospinal fluid
CTD: connective tissue disease
dsDNA: anti-double stranded deoxyribonucleic acid
ELISA: enzyme-linked immunosorbent assay
ENA: extractable nuclear antigens
GPA: granulomatosis with polyangiitis
IgG/M/A: immunoglobulin G/M/A
LP: lumbar puncture
M: measles
MPA: microscopic polyangiitis
MPO: myeloperoxidase
MRI: magnetic resonance imaging
MRZR: MRZ reaction
MRZR: 1 ≥ 1 of 3 AIs positive
MRZR: 2 ≥ 2 of 3 AIs positive
MS: multiple sclerosis
NPSLE: neuropsychiatric systemic lupus erythematosus
n.s: not significant
OCB: oligoclonal bands
OIND: other inflammatory autoimmune neurological diseases affecting the CNS
PPMS: primary progressive MS
PR3: proteinase 3
QAlb: quotient of albumin in CSF/albumin in serum
QIgG: quotient of IgG in CSF/IgG in serum
Qlim: upper limit of the reference range in the CSF/serum quotient diagrams
R: rubella
RDwCNS: rheumatologic disorders with CNS involvement
RRMS: relapsing-remitting MS
SD: standard deviation
SLE: systemic lupus erythematosus
UCTD: undifferentiated connective tissue disease
Z: varicella zoster
